# Comprehensive analysis of transcriptomics and metabolomics to understand tail-suspension-induced myocardial injury in rat

**DOI:** 10.3389/fcvm.2022.1074257

**Published:** 2023-01-17

**Authors:** Yu Liu, Liguo Guo, Chong Xu, Junlian Liu, Quanchun Fan, Yuqing Gai, Shuang Zhao, Xiaorui Wu, Tao Mi, Jiaping Wang, Yongzhi Li

**Affiliations:** China Astronaut Research and Training Center, Beijing, China

**Keywords:** transcriptomic, myocardial injury, bioinformatics, omic analyses, RNA sequencing, tail-suspension, non-targeted metabolomics

## Abstract

**Background/Aims:**

The effect and underlying mechanism of microgravity on myocardium still poorly understood. The present study aims to reveal the effect and underlying mechanism of tail-suspension-induced microgravity on myocardium of rats.

**Methods:**

Tail-suspension was conducted to simulate microgravity in rats. Echocardiography assay was used to detect cardiac function. The cardiac weight index was measured. Hematoxylin and eosin (HE) staining and transmission electron microscopy assay were conducted to observe the structure of the tissues. RNA sequencing and non-targeted metabolomics was employed to obtain transcriptome and metabolic signatures of heart from tail-suspension-induced microgravity and control rats.

**Results:**

Microgravity induced myocardial atrophy and decreased cardiac function in rats. Structure and ultrastructure changes were observed in myocardium of rats stimulated with microgravity. RNA sequencing for protein coding genes was performed and identified a total of 605 genes were differentially expressed in myocardium of rats with tail suspension, with 250 upregulated and 355 downregulated (*P* < 0.05 and | log2fold change| > 1). A total of 55 differentially expressed metabolites were identified between the two groups (VIP > 1 and *P* < 0.05) by the metabolic profiles of heart tissues from microgravity groups and control. Several major pathways altered aberrantly at both transcriptional and metabolic levels, including FoxO signaling pathway, Amyotrophic lateral sclerosis, Histidine metabolism, Arginine and proline metabolism.

**Conclusion:**

Microgravity can induce myocardial atrophy and decreases cardiac function in rats and the molecular alterations at the metabolic and transcriptomic levels was observed, which indicated major altered pathways in rats with tail suspension. The differentially expressed genes and metabolites-involved in the pathways maybe potential biomarkers for microgravity-induced myocardial atrophy.

## Introduction

With the development of the aerospace industry and the increasing duration of space missions, it is essential to understand the effects on human physiology during long-term space travel. Human body has long been adapted to gravity of 1G. When exposed to microgravity, functional and structural changes are occurred in cardiovascular system. It is reported that the microgravity of long-term space flight can cause a series of structural and functional changes in the heart of astronaut, including cardiac remodeling and cardiac dysfunction (1).

The effects of microgravity on heart and its mechanisms have aroused the attention of researchers. Bigard et al. (2) found a decrease in ventricular weight in rats after 21 days of head-down suspension compared with controls. Whereas, the relative ventricle weight/body weight was not affected by head-down suspension (2). Simulated microgravity by head down (−6°) bed rest (HDBR) could impair ventricular repolarization dispersion in volunteers (3). Similar results were found by Palacios et al. (4) that microgravity conditions increased low-frequency oscillations of ventricular repolarization which is the risk factor for repolarization instabilities and arrhythmias.

To further investigate the effects of microgravity on the heart, tail suspension models of rats and mice were established under simulated microgravity condition. And, Zhong et al. (5) found that simulated microgravity induced cardiac remodeling in mice, which may be related to HDAC4, ERK1/2, LC3-II, and AMPK pathways. Liang et al. found that tail-suspension increased heart angiotensin-II production decreased cardiomyocyte size and heart weight, and induced myocardial dysfunction in mice. Whereas, losartan could attenuate microgravity-induced myocardial abnormalities by inhibiting p47 phosphorylation, NADPH oxidase activation and MuRF1 expression (6).

Although the relationship between microgravity conditions and myocardial atrophy has been explored (7, 8). However, the exact underlying mechanisms that microgravity induces myocardial atrophy are not fully understood. The aim of this study was to observe the effects of microgravity on cardiac function and structure in tail-suspension rat model, and analyze the potential molecular mechanisms of myocardial atrophy through RNA sequencing and non-targeted metabolomics.

## Materials and methods

### Animal model construction

Male Sprague–Dawley (SD) rats (6–8 weeks) were obtained from the Laboratory Animal Center of the Chinese People’s Liberation Army Academy of Military Medical Sciences, SCXK-2007-004. Rats were fed in a single cage in the SPF Animal Room of the Chinese Astronaut Research and Training Center. The room temperature was 25°C and the relative humidity was 60%. The rats were kept at 12 h of light/darkness. After 1 week of adaptation, the rats were randomly divided into control group (C group) and tail suspension-induced microgravity group (M group). Then, the rats in the control group were continued feeding in a single cage. And rats in the microgravity group were moved freely in a suspension cage, and the tile was suspended at −30° to simulate weightlessness (9).

### Echocardiography

Echocardiographic examination was performed 4 weeks after tail suspension. The rats were anesthetized with 4% chloral hydrate intraperitoneally (400 mg/kg) and the hair from the chest was removed. The dynamic images of heart were collected, and parapmaers including left ventricular end-diastolic volume (LVEDV), left ventricular posterior wall thickness at end diastole (LVPWd), left ventricular posterior wall thickness at end systole (LVPWs), left ventricular internal diameter at end diastole (LVIDd), interventricular septum thickness at end diastole (IVSd), interventricular septum thickness at end systole (IVSs) were analyzed. The left ventricular mass (LVM) = 1.053 × [(LVIDd + LVPWd + IVSd) 3–LVIDd3] was calculated. Subsequently, left ventricular internal diameter at end systole (LVIDS), interventricular septum thickness at end diastole (IVSD), interventricular septum thickness at end systole (IVSS), end-diastolic volume (EDV), end-systolic volume (ESV), SV (stroke volume) and heart rate (HR) were measured. The calculation formula is as follows: ejection fraction% (EF%) = 100 × (EDV-ESV) ÷ EDV, SV (stroke volume) = EDV–ESV. HR was measured by Aortic Valve model and cardiac output (CO) calculated by calculation formula: CO = SV × HR÷1000.

### Measurement of cardiac weight index

The second day after dynamic images of heart, the rats were weighed and anesthetized by 2% Pentobarbital sodium (30 mg/kg body weight) intraperitoneally. The heart was collected, washed with saline and weighed.

### Hematoxylin and eosin (HE) staining

The heart of rats (no more than 0.5 cm thick) were placed in 10% formalin to maintaining the original morphological structure of tissues. The tissues were dehydrated, embedded in paraffin, and cutted into slices (5–8 μm). Xylene was used to remove paraffin from the slices. Then, the slices were stained with HE staining agents according to the manufacturer’s instructions. After staining, the images of slices were captured under a microscope.

### Ultrastructural examination of myocardium

Left ventricular tissues were taken from rats, with the volume of 1 mm^3^. The tissues were pre-fixed in 3% glutaraldehyde solution for 2 h, then fixed at 4°C for 48–72 h, fixed with 1% osmium tetroxide for 2 h, dehydrated with uranium acetate and citric acid. Lead double staining was observed by transmission electron microscopy.

### RNA sequencing

Ribonucleic acid sequencing was conducted by Oebiotech (Shanghai, China). Raw data were processed using Trimmomatic (10). Using hisat2, the clean reads were mapped to reference genome (11). The read counts aligned to the protein coding genes were obtained by htseq-count (12). The FPKM value of each gene was calculated by cufflinks (13). Differentially expressed genes (DEGs) were identified using DESeq with *P* < 0.05 and | log2FoldChange| > 1 (14).

### Function analysis

The genes mapped to STRING database^1^ were selected to construct Protein–protein interaction (PPI) network (15). Cytoscape software (version 3.9.1^2^) performed the network visualization and analysis (16). Enrichment analysis was performed using DEGs and differentially expressed metabolites (DEMs). Gene Ontology database was used to perform GO analysis (17). Kyoto Encyclopedia of Genes and Genomes (KEGG) database and Gene set enrichment analysis (GSEA) was used to analyze pathways (18, 19). A comprehensive platform MetaboAnalyst was used to further analyze the metabolomics data^3^ (20, 21).

### Sample preparation for metabonomics

Heart tissues from rats were collected and grinded with liquid nitrogen for metabolite extraction. The repeatability and stability of LC-MS analysis was evaluated using quality control (QC) sample. QC sample were prepared by combining of tissue extraction.

### LC-MS based metabonomics analysis

All samples were detected using a Nexera ultraperformance liquid chromatography (UPLC) system combined with a high-resolution tandem mass spectrometer system. An ACQUITY UPLC T3 column (100 mm × 2.1 mm, 1.8 μm; UK) was used for the separation of metabolites. Sample injection volume was 2 μL. The mobile phase consisted of solvent A (water, 0.1% formic acid) and solvent B (Acetonitrile, 0.1% formic acid). The gradient elution conditions were as follows. Flow rate: 0.35 mL/min. 5% B for 0–2 min; 5–100% B for 2–15 min; 100% B for 14–15 min; 100–5% B for 15–15.1 min; and 5% B for 15.1–16 min. The column temperature was maintained at 45°C. The MS conditions were as follows. Mass scan range for positive and negative ion models were both between 125 and 1000. Spray voltage for positive and negative ion models were 3500 and −3000, respectively. Capillary temperature was 320°C. To evaluate the repeatability of LC-MS, QC samples were analyzed randomly.

### Data preprocessing and statistical analysis of metabonomics

The acquired LC-MS raw data was analyzed using Progenesis QI v2.3 (Corporation, Milford, MA, USA). Each ion was identified by data including m/z, peak RT and peak intensities. Ion information was matched to the self-built and public database such as HMDB^4^ and LIPID MAP.^5^ A combined data of positive and negative model was acquired. Principle component analysis (PCA), partial least squares discriminant analysis (PLS-DA), and orthogonal partial least squares discriminant analysis (OPLS-DA) were performed to evaluate variance between two groups. DEMs were identified as VIP > 1 (OPLS-DA model) and *P* < 0.05.

### Statistical analysis

Data are expressed as mean ± SD. Student *t*-test was used to compare difference between two groups. A two tailed *P* < 0.05 was considered statistically significant.

## Results

### Myocardial atrophic changes in rats exposed to microgravity

The average body weight of the rats in the control and microgravity groups were similar before the experiment. The body weight of rats in the microgravity group was decreased. We evaluated the alteration in rat heart weight using cardiac index (the ratio of heart weight to body weight), which eliminated the effect of body weight. The experimental results showed that the heart weight and the cardiac index of the microgravity group were significantly lower than those in the control group, indicating the existence of myocardial atrophy in rats under long time tail suspension simulated microgravity (Table 1). Moreover, we found that the LVM, LVEDV, LVPWd, and LVPWs of rats in the microgravity group were lower than that in the control group (Table 2).

**TABLE 1:** The effect of microgravity on heart weight and cardiac index in rats.

Group	4 weeks heart weight (g)	Heart weight index
Control	1.34 ± 0.08	3.33 ± 0.11
Microgravity	1.02 ± 0.06**	3.05 ± 0.17**

Data are presented as mean ± SD; *n* = 7. **P < 0.01 vs. control group.

**TABLE 2:** The effect of microgravity on ventricular thickness and volume of rats.

	Control	Microgravity
LVM (g)	0.65 ± 0.03	0.39 ± 0.06**
LVEDV (mL)	0.69 ± 0.08	0.50 ± 0.05**
LVPWd (cm)	0.16 ± 0.01	0.14 ± 0.01**
LVPWs (cm)	0.27 ± 0.02	0.24 ± 0.02*

Data are presented as mean ± SD; *n* = 7. *P < 0.05, **P < 0.01 vs. control group. LVM, left ventricular mass; LVEDV, left ventricular end-diastolic volume; LVPWd, left ventricular posterior wall thickness at end diastole; LVPWs, left ventricular posterior wall thickness at end systole.

### The effects of microgravity on cardiac function in rats

After 4 weeks tail suspension, echocardiography was performed to detect the changes of heart function (Table 3). We found that the stroke volume (SV), cardiac output (CO) and ejection fraction (EF) of rats in microgravity group were significantly lower than those of the control group. Fractional shortening (FS) of tail-suspended rats were significantly decreased compared with control rats. Moreover, compared with the control group, left ventricular internal diameter and interventricular septum thickness at end diastole or end systole were decreased in the microgravity group. In contrast, the HR of the rats exposed to microgravity were higher than that of the control rats.

**TABLE 3:** The effect of microgravity on heart function of rats.

	Control	Microgravity
LVIDd (cm)	0.67 ± 0.03	0.58 ± 0.05**
LVIDs (cm)	0.37 ± 0.03	0.30 ± 0.03**
IVSd (cm)	0.14 ± 0.01	0.12 ± 0.01**
IVSs (cm)	0.22 ± 0.02	0.18 ± 0.01**
SV (mL)	0.58 ± 0.07	0.38 ± 0.07**
EF (%)	81.64 ± 1.79	75.54 ± 2.20**
FS (%)	44.57 ± 1.87	40.93 ± 1.57**
HR (BPM)	328.5 ± 17.4	417.8 ± 23.8**
CO (mL)	203.9 ± 9.5	143.6 ± 23.1**

Data are presented as mean ± SD; *n* = 7. **P < 0.01 vs. control group. LVIDd, left ventricular internal diameter at end diastole; LVIDs, left ventricular internal diameter at end systole; IVSd, interventricular septum thickness at end diastole; IVSs, interventricular septum thickness at end systole; SV, stroke volume; EF, ejection fraction; FS, fractional shortening; HR (BPM), heart rate; CO, cardiac output.

### Myocardial histomorphological changes in rats with tail suspension

**FIGURE 1:**
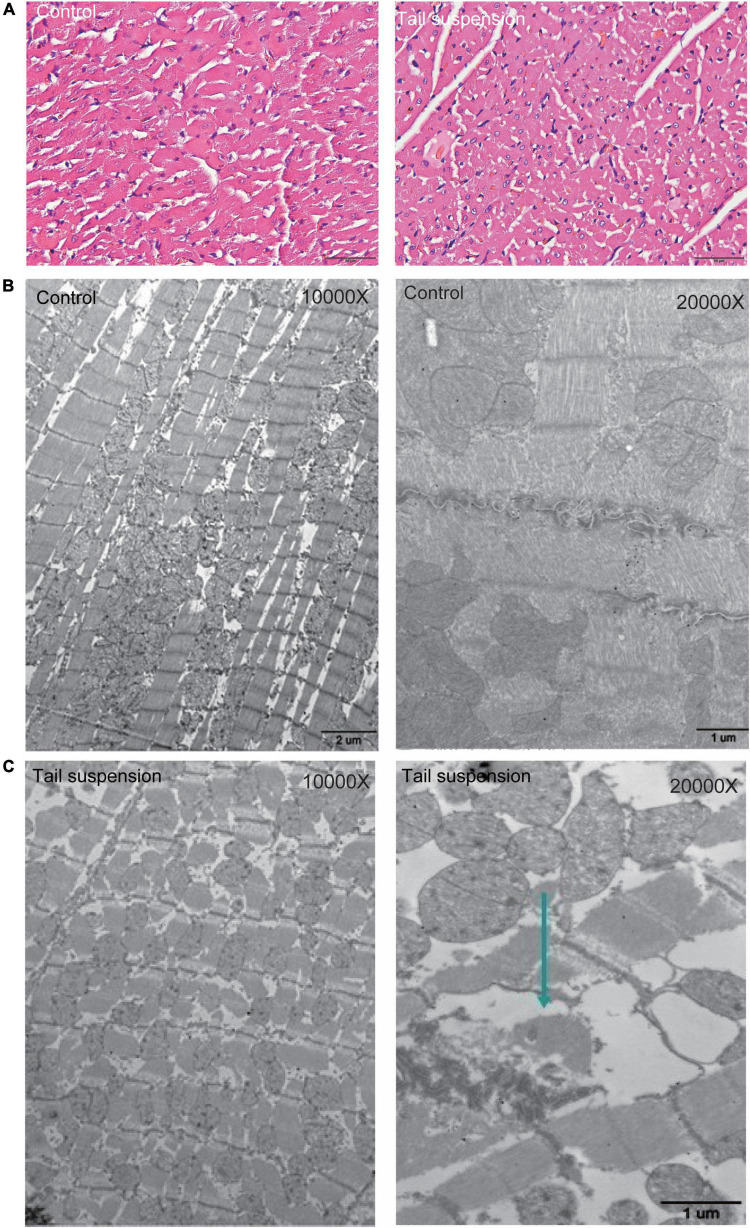
Histological changes in myocardium of rats with tail suspension-induced microgravity. **(A)** Representative hematoxylin and eosin (HE) staining images. **(B)** Representative electron microscope images of rat (10000×). **(C)** Representative electron microscope images of rat (20000×, Arrow represents broken and scattered).

HE staining was used to observe the myocardial structure of rats. The myocardial fibers in the control group were arranged neatly and tightly, and distributed in bundles without fracture. Compared with the control group, the myocardial fibers in the microgravity group were swollen, loose, and fractured. In addition, granular degeneration, vacuolar degeneration, local myocardial fibrotic necrosis and interstitial edema were observed (Figure 1A). We further evaluated the changes of myocardial ultrastructure in rats after 4 weeks tail suspension. The myocardium and sarcomere structures of the control group were intact, and the myofilaments were clear and tightly arranged. Besides, the mitochondrial interstitium was dense, the mitochondrial ridges were clearly visible, and the desmosomes were clear and with complete morphology. After 4 weeks of tail suspension, the myocardial fibers in microgravity group were broken and scattered (Arrow), and the structure of myofibrils was obviously blurred. Mitochondrial ridges were fractured and swollen (Figures 1B, C).

### Differential molecular expression pattern at the transcriptional and metabolic levels in rats with tail suspension

**FIGURE 2:**
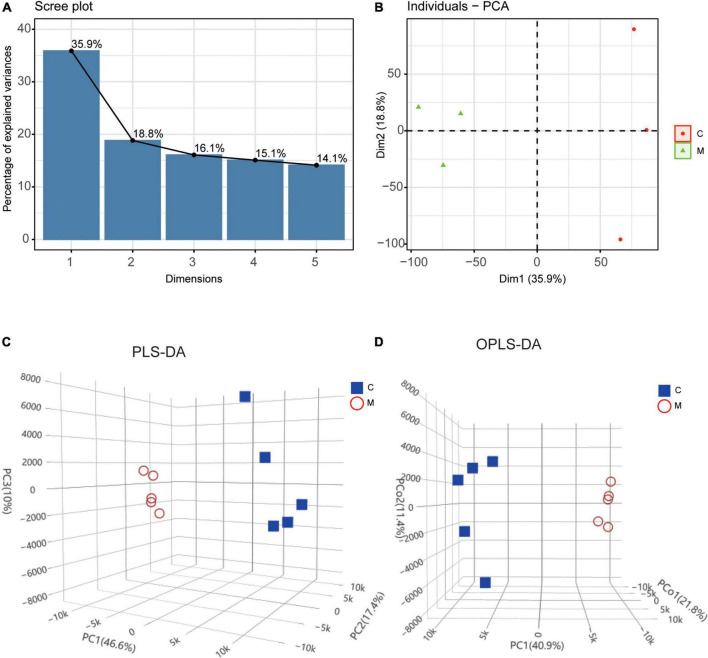
Differential molecular expression pattern at the transcriptional and metabolic levels in rats with tail suspension. **(A)** The scree plot of the principal components in transcriptional dataset. **(B)** PCA and clustering analysis in transcriptional dataset. **(C)** Three-dimensional score plot of samples using partial least squares discriminant analysis (PLS-DA) model. **(D)** Three-dimensional score plot of samples using orthogonal partial least squares discriminant analysis (OPLS-DA) model.

The transcriptomic profiles of control group (*n* = 3) and tail suspension-induced microgravity group (*n* = 3) were acquired by RNA sequencing and the metabolites were detected by UPLC in myocardium of rats with tail suspension (*n* = 5) and control group (*n* = 5). PCA and clustering showed the difference of mRNA expression between the two groups (Figures 2A, B). As for metabolomics, PLS-DA and OPLS-DA were performed to further evaluate the metabolic differences between the two groups and metabolites exhibited different patterns in the two groups (Figures 2C, D).

**FIGURE 3:**
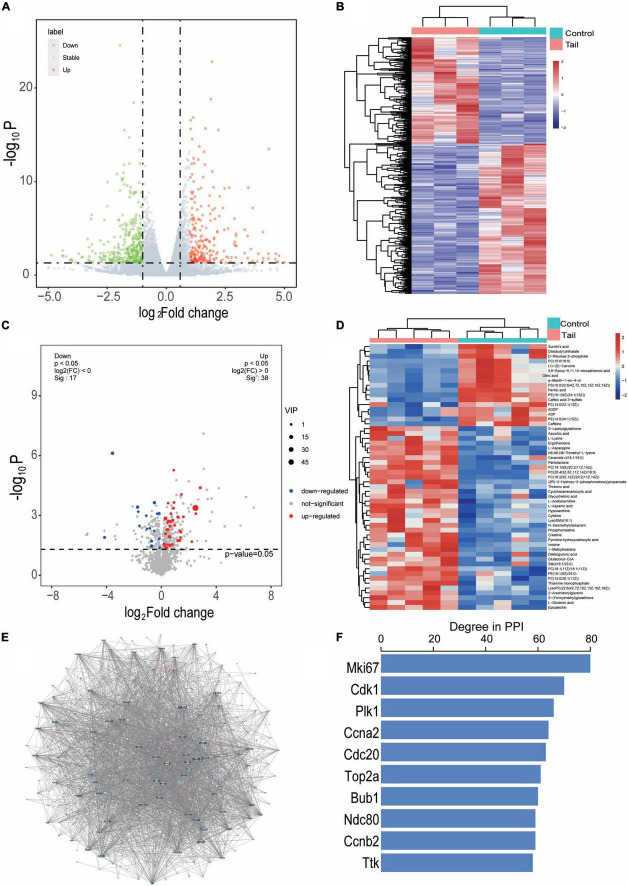
Differentially expressed genes and metabolites in myocardium of tail suspension rats. **(A)** Valconal plots of differentially expressed genes (DEGs, *P* < 0.05 and | log2 FC| > 1). **(B)** Heatmap of DEGs between the two groups. C, control; M, microgravity. **(C)** Volcano plots of differential expressed metabolites (DEMs) between the two groups. **(D)** Heatmap of DEMs between the two groups (VIP > 1 and *P* < 0.05). **(E)** The protein-protein interaction of the DEGs between the two groups, blue represents downregulated, red represents upregulated. **(F)** The top 10 hub genes of degree distribution.

The DEGs with a cutoff value of *P* < 0.05 and | log2 FC > 1| were presented as valconal plots and a total of 605 genes were differentially expressed in myocardium of rats with tail suspension, with 250 upregulated and 355 downregulated (Figure 3A). Hierarchical clustering of the DEGs was showed in Figure 3B. DEMs were identified as OPLS-DA model VIP > 1 and *P* < 0.05. The heatmap and volcano plot of DEMs between the two groups (Figures 3C, D). A total of 55 DEMs were identified between the two groups.

In addition, the PPI of these 605 DEGs were established by STRING including 433 nodes and 2538 interacting pairs (Figure 3E and Supplementary Table 1). By degree analysis by Cytoscape, the top 10 genes named Mki67, Cdk1, Plk1, Ccna2, Cdc20, Top2a, Bub1, Ndc80, Ccnb2, Ttk were considered hub genes in the network (Figure 3F).

### Transcriptomic pathway alterations in myocardium of rats with tail suspension

**FIGURE 4:**
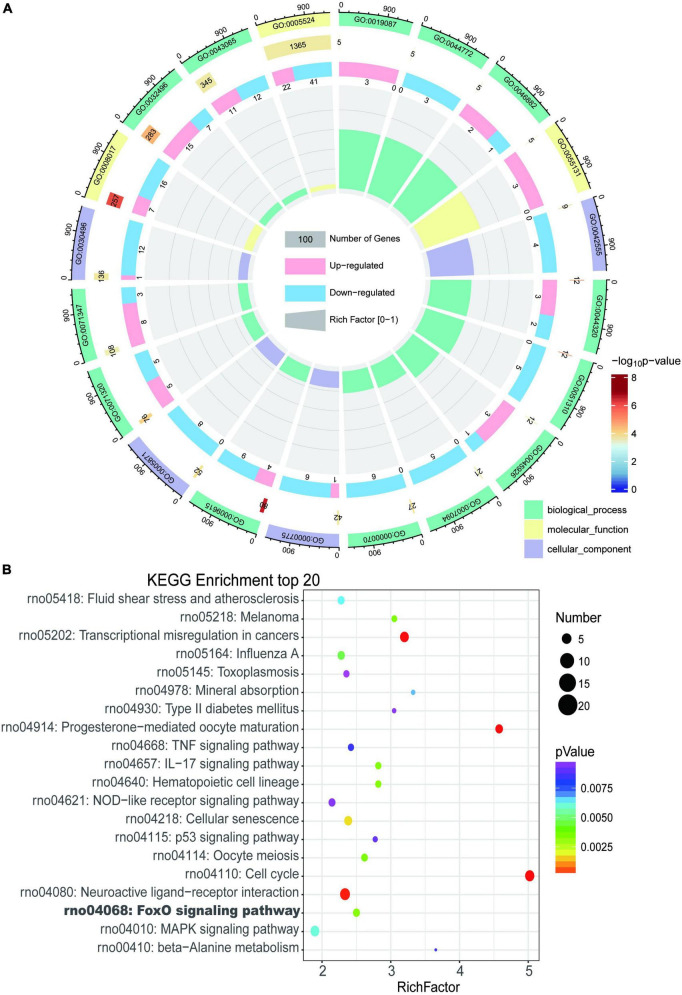
Enrichment analysis of differentially expressed genes in myocardium of tail suspension rats. **(A)** The Circos diagram of top 20 enriched Gene Ontology (GO) terms of differentially expressed genes. **(B)** The Bubble chart of top 20 enriched Kyoto Encyclopedia of Genes and Genomes (KEGG) pathways of differentially expressed genes.

Gene Ontology and KEGG enrichment analysis were performed and we identified 433 enriched GO terms and 48 KEGG pathways based on the above 605 DEGs (*P* < 0.05, Supplementary Table 2). Top 20 GO terms and pathways ranked by *P*-value were in Figures 4A, B respectively.

The most enriched GO terms in BP were “GO:0009615–response to virus” (*P* < 0:001, *n* = 13); in CC was “GO:0042555–MCM complex” (*P* < 0:001, *n* = 4); and in MF were “GO:0005201–microtubule binding” (*P* < 0:001, *n* = 23). The results of KEGG showed that DEGs were highly enriched in heart related pathways including “Cell cycle,” “NOD-like receptor signaling pathway,” “TNF signaling pathway,” “IL-17 signaling pathway,” “MAPK signaling pathway,” and “cellular senescence.”

**FIGURE 5:**
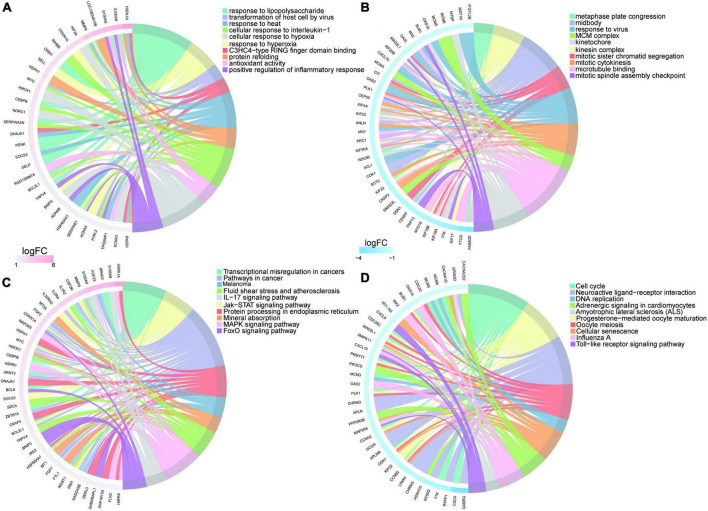
Enrichment analysis of upregulated/downregulated genes in myocardium of tail suspension rats. **(A)** Top 10 Gene Ontology (GO) terms of upregulated genes enrichment analysis. **(B)** Top 10 GO terms of downregulated genes enrichment analysis. **(C)** Top 10 Kyoto Encyclopedia of Genes and Genomes (KEGG) pathways of upregulated genes enrichment analysis. **(D)** Top 10 KEGG pathways of downregulated genes enrichment analysis.

The upregulated genes were top enriched in biological processes including “response to lipopolysaccharide,” and “response to hyperoxia,” and “positive regulation of inflammatory response” (Figure 5A) and heart-related pathways including “IL-17 signaling pathway,” “Jak-STAT signaling pathway,” “protein processing in endoplasmic reticulum,” “FOXO signaling pathway,” and “MAPK signaling pathway” (Figure 5C). The downregulated genes were top enriched in biological processes including “metaphase plate congression,” “mitotic sister chromatid segregation,” and “response to virus” (Figure 5B), and heart-related pathways including “NOD-like receptor signaling pathway,” “Toll-like receptor signaling pathway,” “adrenergic signaling in cardiomyocytes,” “cellular senescence,” and “calcium signaling pathway” (Figure 5D).

### Metabolic pathway alterations in myocardium of rats with tail suspension

**FIGURE 6:**
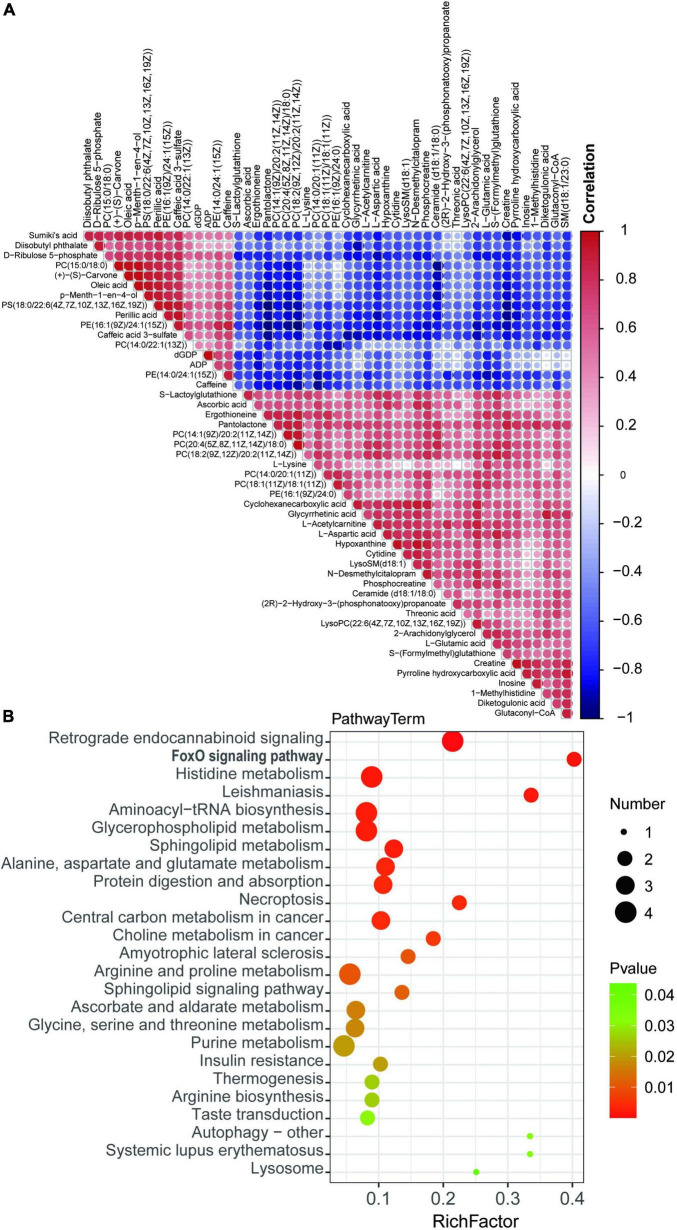
Kyoto Encyclopedia of Genes and Genomes (KEGG) enrichment analysis of differentially expressed metabolites. **(A)** Hierarchical clustering of differential expressed metabolites (DEMs). **(B)** Bubble chart top 20 enriched KEGG pathways of DEMs.

To demonstrate the relationship and metabolites expression among different samples, hierarchical clustering was performed using DEMs. Colors from blue to red indicates the expression abundance of metabolites from low to high (Figure 6A), These metabolites are potential biomarker for cardiac metabolic reprogramming induced by tail suspension in rats. Subsequently, KEGG pathway analysis was performed using DEMs and we identified 25 KEGG pathways. These DEMs were enriched in pathways including “retrograde endocannabinoid signaling,” “FOXO signaling pathway,” “histidine metabolism,” “leishmaniasis,” and “aminoacyl-tRNA biosynthesis,” etc. (Figure 6B and Supplementary Table 3).

### Alterations of major pathways at transcriptional and metabolic in myocardium of rats with tail suspension

**FIGURE 7:**
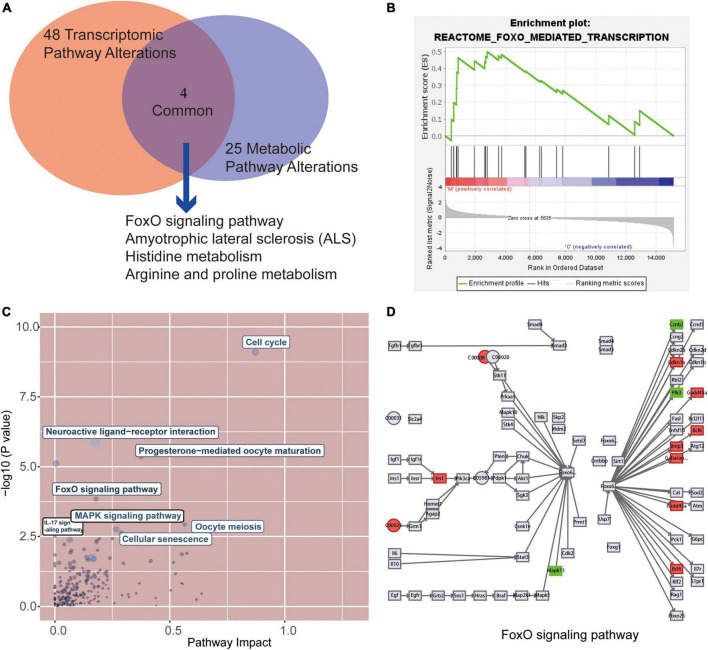
Combined analysis of the alterations pathways at transcriptional and metabolic in myocardium of rats with tail suspension. **(A)** The Venn analysis identified 4 common pathways that were both significantly altered at the transcriptional and metabolic levels in rats with tail suspension. **(B)** Gene set enrichment analysis of FoxO signaling pathway (NES = 1.50, *P* < 0.001). **(C)** Overview of the pathway analysis based on metabolic and transcriptional alteration by MetaboAnalyst. **(D)** Combined analysis of differentially expressed genes (DEGs) and DEGs in FoxO signaling pathway.

After metabolic and gene enrichment analysis, we found 4 significantly altered pathways at both the metabolomic and mRNA expression levels in myocardium of rats with tail suspension (Figure 7A), involving “FoxO signaling pathway,” “Amyotrophic lateral sclerosis,” “Histidine metabolism,” “Arginine and proline metabolism.” Simultaneously, the gene set of the FoxO pathways was also significantly by GSEA analysis (Figure 7B). In addition, we performed the combined analysis of transcriptome and metabolome by MetaboAnalyst and Figure 7C was the visualization of the altered pathways with *p*-values and pathway impact. There were 40 pathways enriched based on the above 605 DEGs and 55 DEMs (Supplementary Table 4, *P* < 0.05) including “Cell cycle,” “Neuroactive ligand-receptor interaction,” “Transcriptional misregulation in cancer,” “Progesterone-mediated oocyte maturation,” “FoxO signaling pathway.” Further analysis found that upregulated genes Bcl6, Bnip3, Cdkn1a, Gabarapl1, Gadd45b, Irs2 and downregulated genes Ccnb2, Mapk11, and Plk1 in microgravity group were enriched in FOXO signaling pathway. Metabolites ADP (C00008) was downregulated and L-glutamate (C00025) was upregulated in microgravity group, which were also enriched in FOXO signaling pathway (Figure 7D).

## Discussion

Aerospace medical research shows that space weightlessness and simulated weightlessness may cause myocardial atrophy and functional changes. Herault et al. (22) studied astronauts who had been flying for 6 months on the Mir space station and found that long-term flight reduced stroke volume. Studies found that LV mass index, LV end-diastolic volume and mean wall thickness decreased, suggesting that the occurrence of myocardial atrophy was an adaptive behavior caused by reduced in myocardial load (23, 24).

The experimental results of this study showed that the left ventricular mass index and the heart weight index decreased in rats with tail suspension, indicating that microgravity caused myocardial atrophy in rats (Table 1). Moreover, the left ventricular mass, wall thickness and end-diastolic volume were reduced in rats with tail suspension (Table 2). This was consistent with the findings of Levine et al. (25) that HDBR and space flight could lead to atrophy of human myocardium. This phenomenon has also been demonstrated in mice (26). At present, there are many studies that are consistent with our findings. Perhonen et al. (24) reported that the left ventricular mass decreased when myocardial atrophy was caused by long-term bed rest and space flight. Bigard et al. (2) reported that the LV weight decreased in tail suspension rats. Therefore, long-term simulated weightlessness causes myocardial atrophy. The microgravity is also proved to affect cardiac function. In this study, we found that the short-axis shortening rate and EF were decreased in long-term simulated microgravity in rats (Table 3). The changes in all these parameters were consistent with the changes in astronauts. Although the heart rate of the rats was increased, the cardiac output was still decreased, indicating that the cardiac function of rats with tail suspension was decreased. Histological changes in myocardium were observed in rats in microgravity group. These findings were consistent with the phenomenon of mitochondrial damage and ridge fracture in rats after 21 days of weightless (27).

To explore the transcriptomics and metabolomics changes in rats with tail suspension and underlying mechanisms of microgravity induced myocardial atrophy, RNA sequencing and non-targeted metabolomics were conducted. Using RNA sequencing, a total of 605 DEGs were identified in myocardium of rats with tail suspension. The hub gens Cdk1, Plk1, Cdc20, Top2a, Ccnb2, and Ttk have been reported that they involved in the myocardial progression (28–33). GO and KEGG enrichment analysis were performed using DEGs. The DEGs were enriched in some pathways that play important role in cardiovascular system, including “NOD-like receptor signaling pathway,” “TNF signaling pathway,” “IL-17 signaling pathway,” “MAPK signaling pathway,” and “cellular senescence,” etc. As for metabol/omics, a metabolism difference was observed between control and microgravity group. A total of 55 DEMs were identified between the two groups (VIP > 1 and *P* < 0.05). These metabolites are potential biomarker for microgravity induced cardiac metabolic reprogramming. KEGG analysis was performed using these altered metabolites. The DEMs were enriched in pathways including “retrograde endocannabinoid signaling,” “FOXO signaling pathway,” “histidine metabolism,” “leishmaniasis,” and “aminoacyl-tRNA biosynthesis,” etc.

Finally, combined analysis was conducted using both transcriptome and metabolome data. Differentially expressed protein coding genes and metabolites were enriched in “Cell cycle, “FoxO signaling pathway,” “Oocyte meiosis,” “MAPK signaling pathway,” “Cellular senescence,” “IL-17 signaling pathway.” FOXO1 plays an important role in diabetic cardiomyopathy (34, 35). Puthanveetil et al. (36) found that the increased nuclear FOXO1 in myocardium of diabetic rats mediates caspase-3 and PARP1-related cell death. In our study, three downregulated DEGs (Ccnb2, Mapk11 and Plk1) and two metabolites (ADP and L-glutamate) were enriched in FOXO pathway. Mapk11 encoded p38 mitogen-activated protein kinase paly pivotal pathophysiological role in diabetic cardiomyopathy, cardiac ischemia/reperfusion injury, and cardiac hypertrophy (37, 38). Calpain activation participates microgravity-induced myocardial abnormalities in mice, which was through p38 and EERK1/2 MAPK (26). Ccnb2 (Cyclin B2) is a cell cycle related gene, which is associated with cancer progression and prognosis (39–41). Polo-like kinase 1 encoded by Plk1 is a negative regulator of FOXO3 (42). However, little is known about the role of Ccnb2 and Plk1 in cardiomyocytes. The altered metabolites ADP (downregulated) and L-glutamate (upregulated) in microgravity group were enriched in FOXO signaling pathway. Glutamate has the potential of increase heart rate and induce cardiac fibrosis (43, 44). ADP has protective effect against cardiac mitochondria injury under severe oxidative stress (45). Therefore, the DEGs and DEMs-involved in this pathway maybe potential biomarkers for microgravity-induced myocardial atrophy.

In conclusion, microgravity induces myocardial atrophy and decreases cardiac function in rats and the transcriptomics and metabolomics were observed alterations in rats with tail suspension. This study provides new insights in the systems-level investigation and understanding of the underlying molecular alterations of microgravity induced myocardial injury that could facilitate the development of novel therapeutic targets and biomarkers for the disease.

## Data availability statement

The datasets presented in this study can be found in online repositories. The names of the repository/repositories and accession number(s) can be found in the article/Supplementary material. RNA sequencing expression profile and non-targeted metabolomics data were provided in the Supplementary Tables 5, 6.

## Ethics statement

The experimental procedure was pre-approved by China astronaut research and training center, China (No. 100094).

## Author contributions

JW and YZL contributed to the conception and design of the study. YL, LG, CX, and JL wrote or contributed to the writing of the manuscript. YL, QF, YG, SZ, XW, and TM did animal experiment and the bioinformatic analysis. All authors reviewed the manuscript.

## Footnotes

1
https://cn.string-db.org/

2
https://cytoscape.org/

3
https://www.metaboanalyst.ca/MetaboAnalyst/

4
http://www.hmdb.ca/

5
http://www.lipidmaps.org

## Supplementary material

Supplementary Table 1 The Protein–protein interaction (PPI) of these differentially expressed genes (DEGs) established by STRING.

Supplementary Table 2 Gene Ontology (GO) and Kyoto Encyclopedia of Genes and Genomes (KEGG) enrichment analysis of these differentially expressed genes (DEGs).

Supplementary Table 3 Kyoto Encyclopedia of Genes and Genomes (KEGG) pathway analysis was performed using the differential expressed metabolites (DEMs).

Supplementary Table 4 The combined analysis of transcriptome and metabolome by MetaboAnalyst.

Supplementary Table 5 RNA sequencing was employed to obtain transcriptome expression profile of heart from tail-suspension-induced microgravity and control rats.

Supplementary Table 6 Non-targeted metabolomics was employed to obtain metabolic signatures of heart from tail-suspension-induced microgravity and control rats.
